# Supplementary material for: Musashi binding elements in Zika and related Flavivirus 3′UTRs: A comparative study in silico
Source: Sci Rep. 2019 May 6;9:6911. doi: 10.1038/s41598-019-43390-5 (PMC6502878; doi:10.1038/s41598-019-43390-5)
Supplement: Supplementary file 1 — Supplementary Data [file 41598_2019_43390_MOESM1_ESM.pdf]

Musashi binding elements in Zika and related Flavivirus 3'UTRs: A comparative study *in silico*
# - Supplementary Material -

Adriano de Bernardi Schneider [1] and Michael T. Wolfinger [2*]

[1] Department of Medicine, University of California San Diego, 220 Dickinson St, Suite A, San Diego, CA 92103, United States of America
[2]Department of Theoretical Chemistry, University of Vienna, Währingerstraße 17, 1090 Vienna, Austria

1 Pentanucleotide accessibility in ZIKV 3'UTR

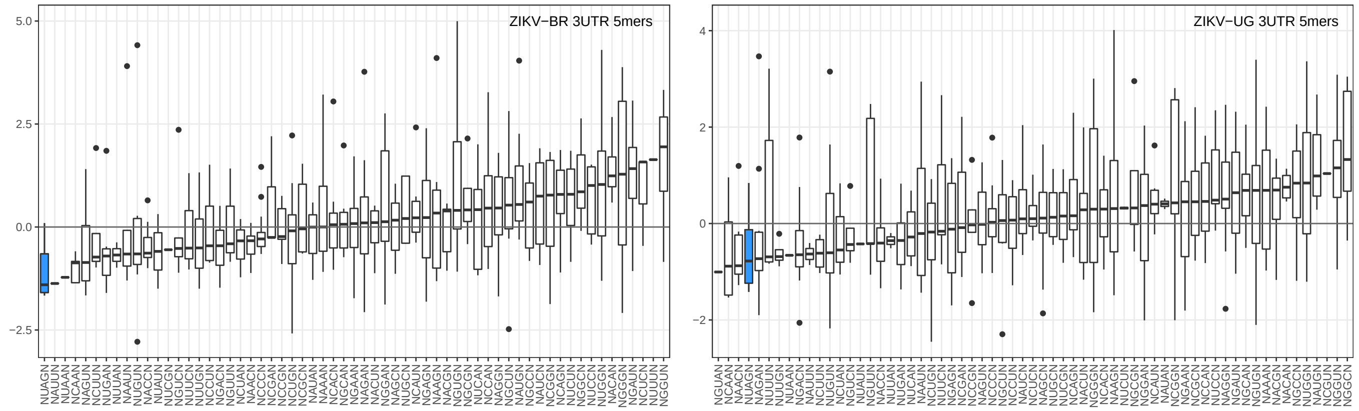

Figure 1: Distribution of z scores of opening energies for pentanucleotides found in the 3'UTR of ZIKV from Brazil (left) and Uganda (right), sorted by median opening energy z score. Each bin comprises data for all pentanucleotides $NXYZN$ that have a central trincleotide XYZ enclose by arbitrary nucleotides N. Pentanucleotides $NUAGN$ (highlighted in blue) have low opening energy z scores, i.e. they are highly accessible in the structural ensembles of both ZIKV lineages.

2 Spondweni virus 3'UTR

The downloaded `refseq` genome for Spondweni virus (SPONV) `NC_029055.1` does not include the 3'UTR sequence. Since SPONV is phylogenetically related to Zika virus (ZIKV) [1], we were looking to include this sequence into our analaysis.

Nikos Vasilakis (Univ. of Texas Medical Branch, Galveston, TX) generously provided SPONV sequences. The 338 nt 3'UTR sequence of the SA-Ar strain (listed below) has been added to the set of flavivirus sequences analyzed in this study.

```
>SPONV SA-AR|3UTR|
AUAAUGUAAAUAAUAAAUAAAUUAAUAAAGUAAGGAUAGGAAACUAACCUAGCCUAACUA
ACAAAGUCAGGCCGUAGAUUAAGACGCCAUGGCACGGAAGAAGCCAUGCUGCCUGUGAGC
CCCCCAGGAGGAUCUGGGUUAACAAAGAGAGCAAUGUCUCUCCCACGCCUGGAAGAGGUG
GCGAUCUCUCCAGAGCGGUAAAAGCGUGGGGCCUGAAGGCAGGAGGAGCUGUGACUCCUG
CUGGAGGGACUAGCGGUUAGAGGAGACCCCCCACAAAACGCAAAACAGCAUAUUGACGCU
GGGAAAGACCAGAGACUCCGUGCGUUUCCAGCACGCCG
```

*To whom correspondence should be addressed. Email: michael.wolfinger@univie.ac.at

References

[1] Andrew D Haddow, Farooq Nasar, Hilda Guzman, Alongkot Ponlawat, Richard G Jarman, Robert B Tesh, and Scott C Weaver. Genetic Characterization of Spondweni and Zika Viruses and Susceptibility of Geographically Distinct Strains of *Aedes aegypti*, *Aedes albopictus* and *Culex quinquefasciatus* (Diptera: Culicidae) to Spondweni Virus. *PLoS Neglect Trop D*, 10(10):e0005083, 2016.
